# Effect of hot smoking treatment in improving Sensory and Physicochemical Properties of processed Japanese Spanish Mackerel *Scomberomorus niphonius*

**DOI:** 10.1002/fsn3.1715

**Published:** 2020-06-11

**Authors:** Md. Abdul Baten, Na Eun Won, Md. Mohibbullah, Sung‐Joon Yoon, Jae Hak Sohn, Jin‐Soo Kim, Jae‐Suk Choi

**Affiliations:** ^1^ Department of Fishing and Post Harvest Technology Sher‐e‐Bangla Agricultural University Dhaka Bangladesh; ^2^ Seafood Research Center IACF Silla University Busan Korea; ^3^ Department of Food Biotechnology Division of Bioindustry College of Medical and Life Sciences Silla University Busan Korea; ^4^ EBADA Fishery Co. Ltd Busan Korea; ^5^ Department of Seafood and Aquaculture Science Gyeongsang National University Tongyeong‐si Korea

**Keywords:** hot smoking, Japanese Spanish Mackerel, physicochemical properties, processing and preservation, sensory evaluation

## Abstract

Japanese Spanish Mackerel (JSM) *Scomberomorus niphonius* (Cuvier 1832) is an important commercial fish species in South Korea. The postharvest handling, preservation, and storage of JSM have not been clearly understood, and therefore, it is very often oxidized to produce off‐flavor while marketed as the raw or frozen state. To overcome these problems, the present study was designed to adapt the hot smoke processing technique for improving the sensorial, physicochemical, and microbial qualities of JSM with extended shelf life. The hot smoking (70°C) with different sawdusts at the different smoke times (0, 20, 25, and 30 min) was applied to process JSM fillet. The smoked JSM obtained higher sensory attributes (appearance, odor, taste, color, texture, and overall preferences) and suppressed bacterial growth, pH, volatile base nitrogen, thiobarbituric acid‐reactive species, and trimethylamine N‐oxide at an optimum smoking time of 25 min using oak sawdust. Moreover, it possessed higher nutritional value and beneficial polyunsaturated fatty acids such as docosahexaenoic acid (DHA), 4.19 g/100 g, and eicosapentaenoic acid (EPA), 1.82 g/100 g. The smoked JSM product extended shelf life up to 42 days at 10°C storage temperature. The overall findings indicate that the hot smoking technology with JSM could be effective in achieving good sensorial, nutritional, and functional attributes to the consumer.

## Graphical abstract

Effect of sensory evaluation (odor and overall preference) of JSM product using wood smoke of different sawdusts such as apple, chestnut, oak, cherry, and walnut at two different smoking times.

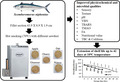

## 1 INTRODUCTION

Japanese Spanish mackerel is an important species of scombrid family (Scombridae) and widely distributed in East Asia such as South Korea, China, and Japan (Nishikawa, Nakamura, & Katayama, 2014). It is reported that approximately 400,000 tonnes of Japanese Spanish mackerel (Seerfish) were caught during the period of 2000 to 2009 (FAO, 2012). It is considered to be one of the highly important commercial fish species in South Korea. This species of mackerel is marketed as raw fish or as processed fish like frozen fillet, salted, dried, and smoked fish.

There is a wide variety of fish preservation techniques, and of which, smoking is one of the oldest methods of fish preservation (Swatawati, Suzuki, & Dewi, 2000). Smoking is a combination of salting, drying, and heating of fishery products (Adeyeye, 2019). Smoking preservation method is the control of physicochemical parameters such as pH level, VBN level, TBARS level, fatty acid content, and texture profile and improves sensory quality, subsequently ensuring the extended storage time of final fishery products with high quality (Huang et al., 2019). Smoked food inhibits microbial growth and delays the oxidative changes (Fuentes et al., 2010). The smoke from incomplete combustion of wood or sawdust allows releasing of volatile chemical compounds, which helps to inhibit the bacterial growth in the processed fish foods when deposited on its surface (Sutikno et al., 2019). The smoked products have considerable consumer demand because of its unique color and flavor. The wood or sawdust used for smoking has released various complex compounds including phenols, ethers, esters, hydrocarbons, acids, alcohols, and ketones, which are responsible for the development of color and flavor in food products (Bashir, Kim, An, Sohn, & Choi, 2017; Goulas & Kontominas, 2005; Guillén & Errecalde, 2002). On the basis of temperature, smoking can be divided into three types: cold (12–25°C), warm (25–45°C), and hot smoking (40–100°C) (Stołyhwo & Sikorski, 2005). Therefore, the present study was designed to optimize and fix the hot smoking (70°C) treatment in the processing and preservation of Japanese Spanish mackerel.

The hot‐smoked products are much better than that of cold‐smoked products in terms of color and appearance (Huang et al., 2019). The hot‐smoking method increases the nutritional composition that may be due to the decreased moisture content of fishery products (Ahmed, Ali, Kalid, Taha, & Mahammed, 2010). The application of hot smoking technique with a combination of 5% brine solution in tilapia can be stored safely under refrigerated conditions for over 35 days (Yanar, Celik, & Akamca, 2006). The heating process is probably the most favorable method to keep the quality of a food product healthy; however, to some extent, heating may cause protein denaturation of food products, which concomitantly decreases in both nutritional and functional attributes (Sutikno et al., 2019). Therefore, it is necessary to optimize the time, temperature, and sawdust material in hot smoking technique to obtain the premium quality of a smoked product. It is evident that the quality attributes of smoked fishery product are determined by various analysis methods including sensory evaluation (color, texture, odor, flavor, and overall acceptance), physicochemical assessment (i.e., pH level, VBN level, TBARS level, TMAO, and fatty acid content), and microbial growth quantification (Mohibbullah, Won, et al., 2018). To date, so far, there are no studies working on the effect of hot smoked Japanese Spanish mackerel in the aspects of processing, preservation, and shelf life. Therefore, the present study aimed to evaluate the effects of hot‐smoked processing technique on sensory, physicochemical, and microbiological qualities of JSM. In addition to these, this study has provided additional insight into favorable effects on the extension of shelf life of processed JSM with remarkable acceptability to the consumer.

## 2 MATERIALS AND METHODS

### 2.1 Collection and Preparation of Sample

The Japanese Spanish mackerel (JSM) is also known as Japanese Seerfish. The collected fish fillet sample section diameter was 43.9 × 8.9 × 1.9 cm, and the average weight of the sample was 448.6 ± 56.6 g.

### 2.2 Smoking with different sawdust

The collected JSM fish fillet was dipped into a brine solution containing 8% NaCl at a ratio of 1:1 (w/w) for 24 hr. The samples were dried to drain off excess water by keeping the samples at 30°C for 30 min. Then, the sample was transferred to the smoke chamber (Braai Smoker, Bradley, Canada) for smoking treatment and the smoke was generated by the combustion of different sawdust (Apple, Chestnut, Oak, Cherry, and Walnut) at 70°C at different time intervals such as 20, 25, and 30 min. The final smoked JSM fish fillet samples were kept at 4°C for further experiments.

### 2.3 Sensory evaluation

The sensory evaluation was performed in treated and untreated smoked products. The evaluated parameters were color, odor, flavor, and overall acceptance. All of the samples were unbiasedly encoded prior to sensory evaluation. For the performance of the sensory test, ten panelists were selected (range of age 25 to 40) and trained them on the evaluation method. The hedonic scale method 1 to 9 (1 for extremely dislike and 9 for extremely like) was used by panelists for scoring the products, and the value 5 was considered as threshold limit and the sample value less than score 5 was considered as rejected (Li, Wang, Fang, & Li, 2013).

### 2.4 Weight loss

The weight loss of JSM the fillet was calculated by the differences in weight before and after oven drying as followed by the method of Goulas and Kontominas (2005).

### 2.5 Total bacterial count (TBC)

The total bacterial count was determined by the serial dilution method. The 1 gm of fish sample was taken and mixed with 9 ml sterile saline solution and then homogenized by using Stomacher 400 Circulator (Seward Limited, West Sussex, UK). Then, the sample was spread onto plate count agar (Difco, Franklin Lakes, NJ, USA) and incubated at 37°C for 48 hr, as followed by Chen, Wu, and Pan (2015). The US Food and Drug Administration (FDA) preferred method was used for the estimation of total coliform. The homogenate sample was inoculated onto EC medium and incubated at 37°C for 24 hr and then observed gas production. If no gas production was found, indicating that the result was considered to be negative (−).

### 2.6 Odor intensity

According to Macagnano et al. (2005), 5 g of sample was taken in 50‐ml conical tube, which fits the applied odor concentration meter (XP‐329, New Cosmos Electric Co. Ltd., Osaka, Japan). Upon closing the lid, the odor intensity was observed until the signal reached a peak point, which expressed as an arbitrary unit.

### 2.7 Color evaluation

The color of the sample was evaluated using a CM‐700d Konica Minolta (Tokyo, Japan) instrument. In compliance with the previous reports (Bashir et al., 2018; Chen et al., 2015), a Hunter system value was used to determine the color of the surface of each sample.

### 2.8 Texture analysis

The Brookfield Texture Analyzer (Massachusetts, USA) was used for the determination of JSM fillet texture. This instrument is operated by computer software (Texture PRO CT, Middleboro, USA). The aluminum cylinder probe with a diameter of 10 mm at 0.5 mm/s cross‐head speed was used to compress 50% of sample height, and it was continued up to 60 s and then extruded. During this period, a number of attributes were measured following the study of Ganesan and Benjakul (2014).

### 2.9 pH measurement

The JSM homogenized fish sample (4 g) was mixed with 45 ml of distilled water (DW) for 2 min and centrifuged (SHG‐15D, SciLab, Seoul, Korea). The supernatant was collected and filtered through Whatman filter paper (Advantec Toyo Kaisha, Ltd., Tokyo, Japan). The pH meter (OHAUS STARTER 2100, Seoul, Korea) with a glass electrode was kept into the homogenate and monitored for measuring the pH of the sample.

### 2.10 Volatile basic nitrogen (VBN)

The Conway microdiffusion method was used to determine the VBN level produced by JSM. A total of 5 g of fish sample was mixed with 25 ml of DW in a glass beaker and homogenized. After filtration, 1 ml of sample solution and 1 ml of potassium carbonate (1 ml) were taken together and mixed up to the outer chamber, and subsequently, 1 ml of 0.1 N HCl was added to the inner chamber of Conway unit. After that, the incubated Conway cell was titrated with 0.01 N NaOH until the color changed (Ali, Hossain, Rashed, Khanom, & Sarower, 2013).

### 2.11 Thiobarbituric acid‐reactive species (TBARS)

The 5 g of sample was homogenized with 12.5 ml of TBARS solution containing 20 trichloroacetic acid and 2 M phosphoric acid, followed by filtration and incubation at 95°C for 30 min (Oğuzhan Yildiz, 2015). Then, 200 μl of each sample was added to each well of the 96‐well plate and recorded the absorbance value at 530 nm wavelength using a nano‐SPECTRO star (Newtown, UK).

### 2.12 TMAO analysis by Gas chromatography‐mass spectrometry (GC/MS)

The GC/MS analysis was used to evaluate the content of trimethylamine N‐oxide (TMAO) in both smoked and nonsmoked JSM samples, as followed by Mohibbullah, Won, et al. (2018). In brief, the 10 g of sample was taken in a 50‐ml falcon tube with 10 ml of DW and then sonicated for 20 min. After centrifugation at 2,200 × *g* for 10 min, the supernatant was filtered and transferred to solid‐phase microextraction system and then volatilized using GC instrument (Agilent 7890B GC) at an oven temperature of 240°C which increased from 40°C to 210°C following the flow rate of 10°C/min. The helium was used as the carrier gas and mixed with the gaseous compounds of the sample, which was separated through a DB‐WAX column (30 m length × 0.25 μm i.d.; 0.25 μm thickness). The identification and quantification of TMAO in nonsmoked and hot‐smoked JSM were confirmed and validated by comparing with that of the standard compound (Sigma‐Aldrich, St. Louise, MO, USA).

### 2.13 Nutritional composition analysis

The nutritional value of JSM was determined using the standard method of AOAC (2000) at Traditional Microorganism Resources Center, Keimyung University, Daegu, Korea. The proximate compositions were considered to be the total content of calories, sodium, carbohydrate, sugars, crude fat, trans fat, saturated fat, cholesterol, crude protein, potassium, calcium, iron, and vitamin D of the selected JSM fillet sample.

### 2.14 Fatty acid analysis by gas chromatography

The hydrolytic method was used to extract fatty acid from JSM fish. The ether was used for extraction, and subsequently, methyl was added to form fatty acid methyl esters (FAMEs). Gas chromatography (Shimadzu Corp., Kyoto, Japan) method was used for the quantification of FAMEs. The FAMEs samples were separated with Supelco SP‐2560 column (100 m × 0.25 mm × 0.25 μm) using an oven temperature of 240°C, which increased from 100°C to 200°C at a flow rate of 3.5°C/min. The carrier gas was helium at a split ratio of 1:50. The identification and quantitation of FAME samples were done by comparing the retention time with fatty acids standard (Sutikno et al., 2019).

### 2.15 Statistical analysis

The data were triplicated and calculated as mean ± standard deviation (std). Analysis of variance (ANOVA) was performed, and the results were considered to be statistically significant at 95% where the *p‐value* is < 0.05. All of the analysis was conducted using IBM SPSS software version 21.0 (IBM, Corp., New York, USA).

## 3 RESULTS

### 3.1 Effect of different sawdust smoke on sensory evaluation

**FIGURE 1:**
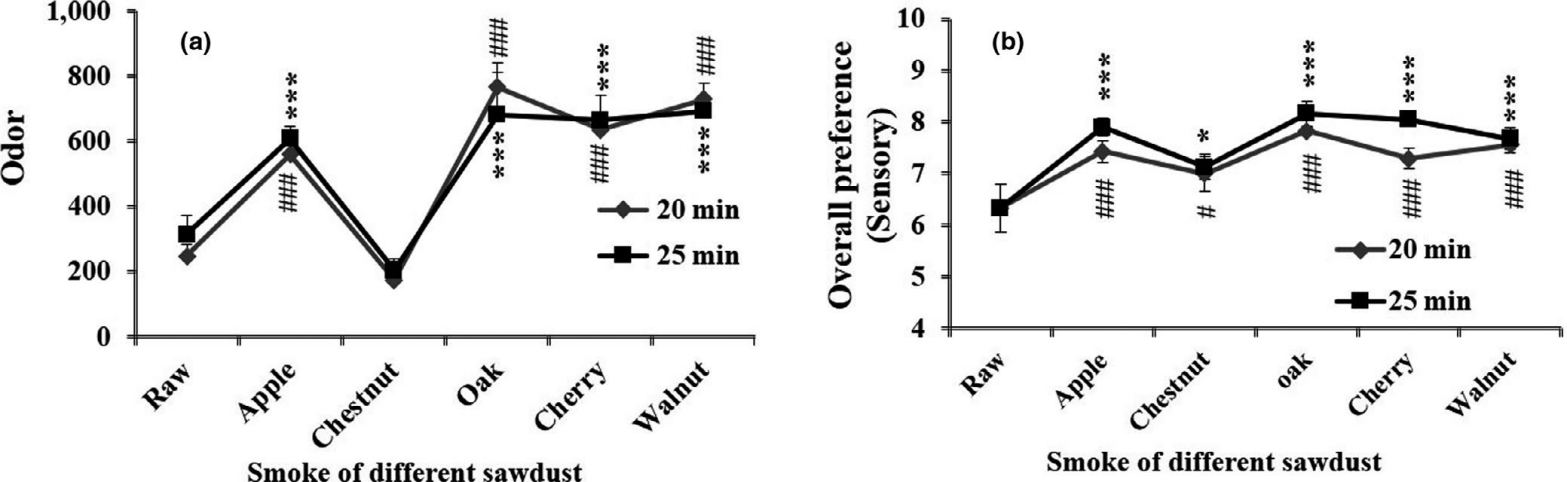
Effect of time‐dependent smoking (0, 10, 15, 20, 25, and 30 min) on instrumental odor and sensory quality attributes (appearance, odor, taste, and overall preference) of hot‐smoked JSM products. Data were expressed as mean ± std (*n* = 10), and statistical significant level was *p < .05, **p < .01, and ***p < .001 (ANOVA)

The different sawdust was used in hot smoking of JSM for the development of color and flavor. The results revealed that a significant (*p* < .001) and good quality sensorial characteristics were observed in processed JSM while using Oak sawdust followed by cherry, apple, walnut, and chestnut sawdusts, at 20 and 25 min smoke time, respectively (Figure 1). We found Oak sawdust was the best smoking materials to be used for improving the quality of JSM product.

### 3.2 Effect of time‐dependent smoking on odor and sensory evaluation

**FIGURE 2:**
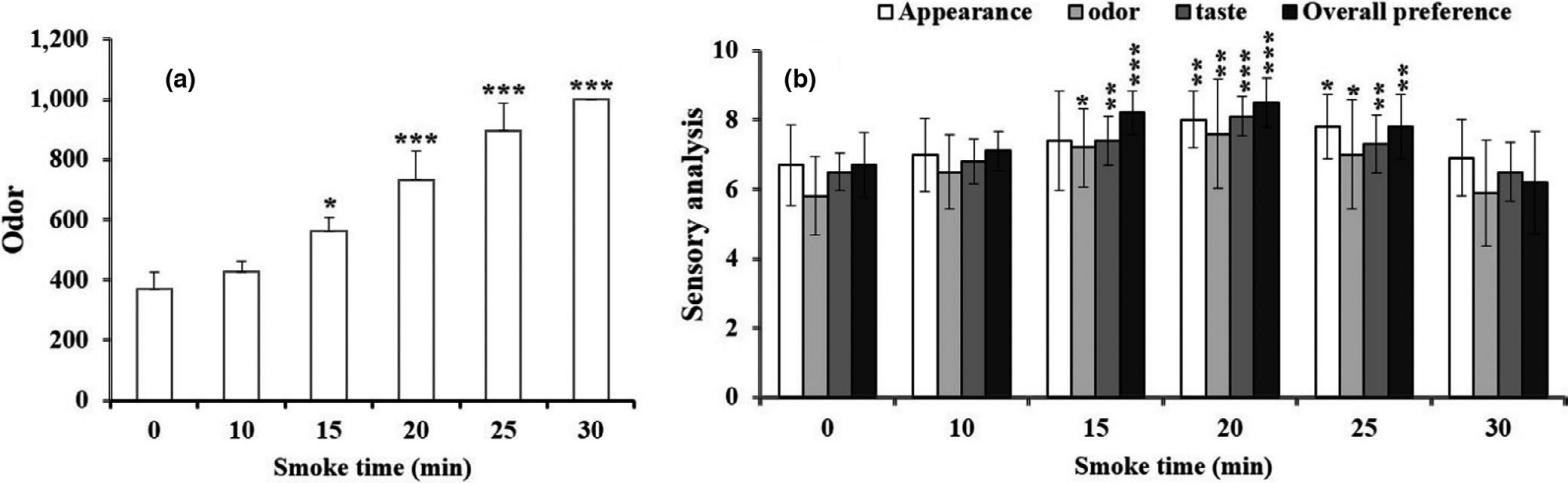
Effect of sensory evaluation (odor and overall preference) of JSM product using wood smoke of different sawdust such as apple, chestnut, oak, cherry, and walnut at two different smoking times (20 and 25 min). Data were expressed as mean ± std (*n* = 10), and statistical significant level was *p < .05, **p < .01, and ***p < .001 (ANOVA)

In order to optimize the smoking time, the instrumental odor analysis and 9‐point hedonic scale analysis were set to perceive the consumer preferences and acceptability of the processed JSM in our study. The time‐dependent smoke time (0 to 30 min) revealed that odor intensity significantly (*p* < .001) increased with the extension of smoking time. The sensory attributes such as appearance, odor, taste, and overall preference had been considered for quality assessment, and in the present study, the results revealed that, among all different smoking time (0 to 30 min), at 20 min achieved higher sensory quality attributes followed by 15 and 25 min (Figure 2). Accumulated results indicated that optimum time for the smoking of JSM was to be at 20 and 25 min, in which odor intensity and sensory attributes were greatly satisfied at those conditions and optimized for further experiments.

### 3.3 Effect of hot smoking on physical properties of JSM

#### 3.3.1 Effect of smoking time on weight loss

**FIGURE 3:**
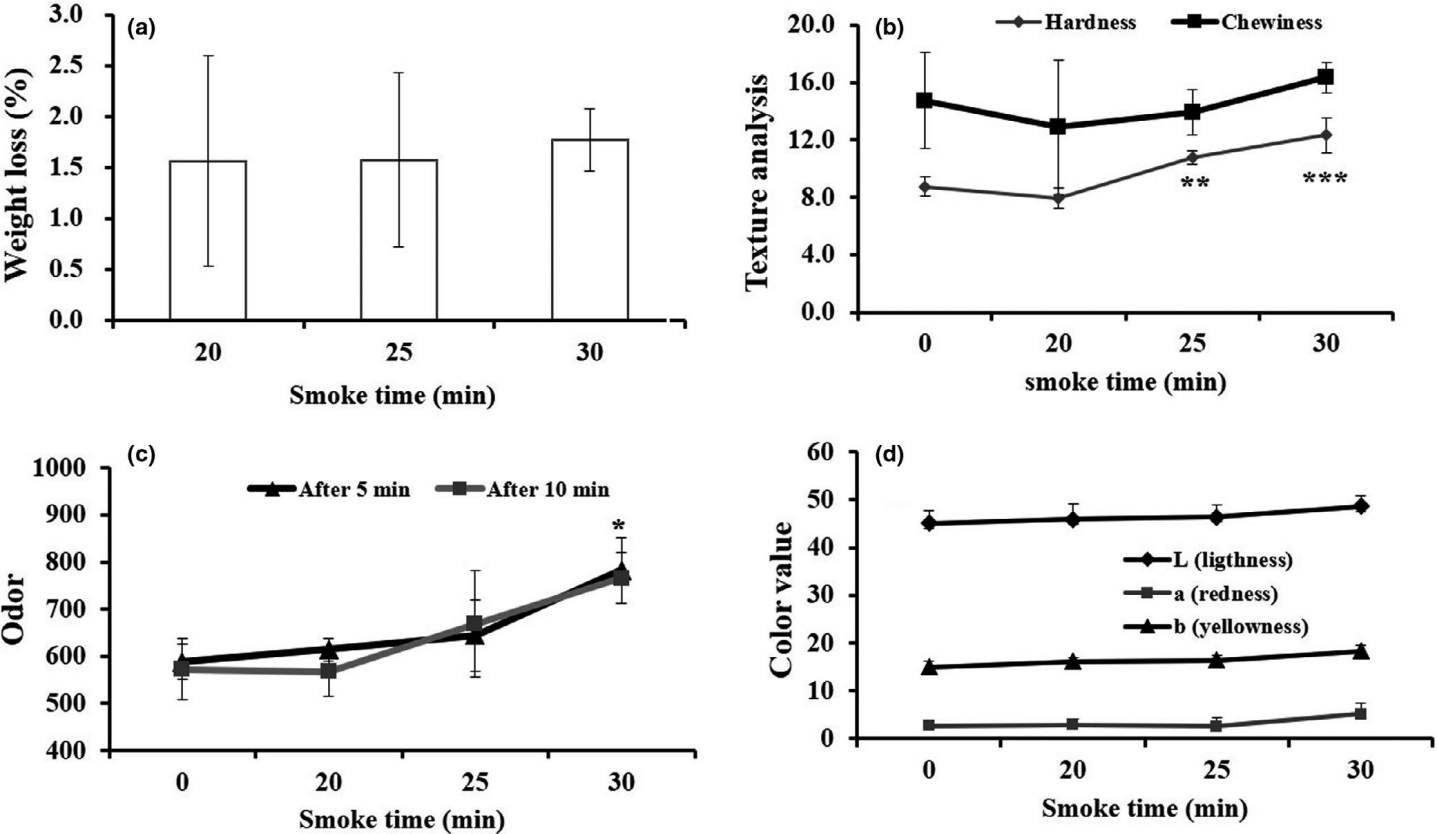
Effect of the best smoke time on weight loss (a), texture analysis (b), odor (c), and color value (d) in hot‐smoked JSM products. Data were expressed as mean ± std, and statistical significant level was expressed as *p < .05 and **p < .01, compared with no treatment group (ANOVA)

The study was observed no significant difference in a weight loss of JSM at the different smoking time from 20 to 30 min (Figure 3a), thus delaying the release of moisture from the fish sample, which may help to retain the firmness of JSM during storage conditions.

#### 3.3.2 Effect of smoking time on texture

Texture analysis of food consisting of hardness and chewiness is one of the important factors to perceive the consumer liking and acceptability of food structure. Here, the hardness of the JSM was consistent with different smoking time but chewiness was found to have significantly (*p* < .01, .001) different with increasing smoking time at 25 and 30 min (Figure 3b).

#### 3.3.3 Effect of smoking time on odor

The odor intensity plays an important role in the product preferences and acceptability by the consumers. The odor intensity of JSM fillet was calculated (after 5‐ and 10‐min interval) and increased with the increase of smoking time from 0 to 30 min (Figure 3c). The result identified the increased level of odor intensity in JSM product while smoking at 30 min. As the increased smoking time declines the acceptability of fishery products, 30‐min smoking might have the negative effect of over combustion of sawdust.

#### 3.3.4 Effect of smoking time on color

The human visual sense is exclusively working on the decision of taking the right food by the consumer. The results showed that the color (lightness, redness, and yellowness) of the hot‐smoked JSM was found to be constant during smoking treatment for 0 to 30 min (Figure 3d).

### 3.4 Effect of hot smoking on biochemical properties of JSM

#### 3.4.1 Effect of smoking time on TBC

**FIGURE 4:**
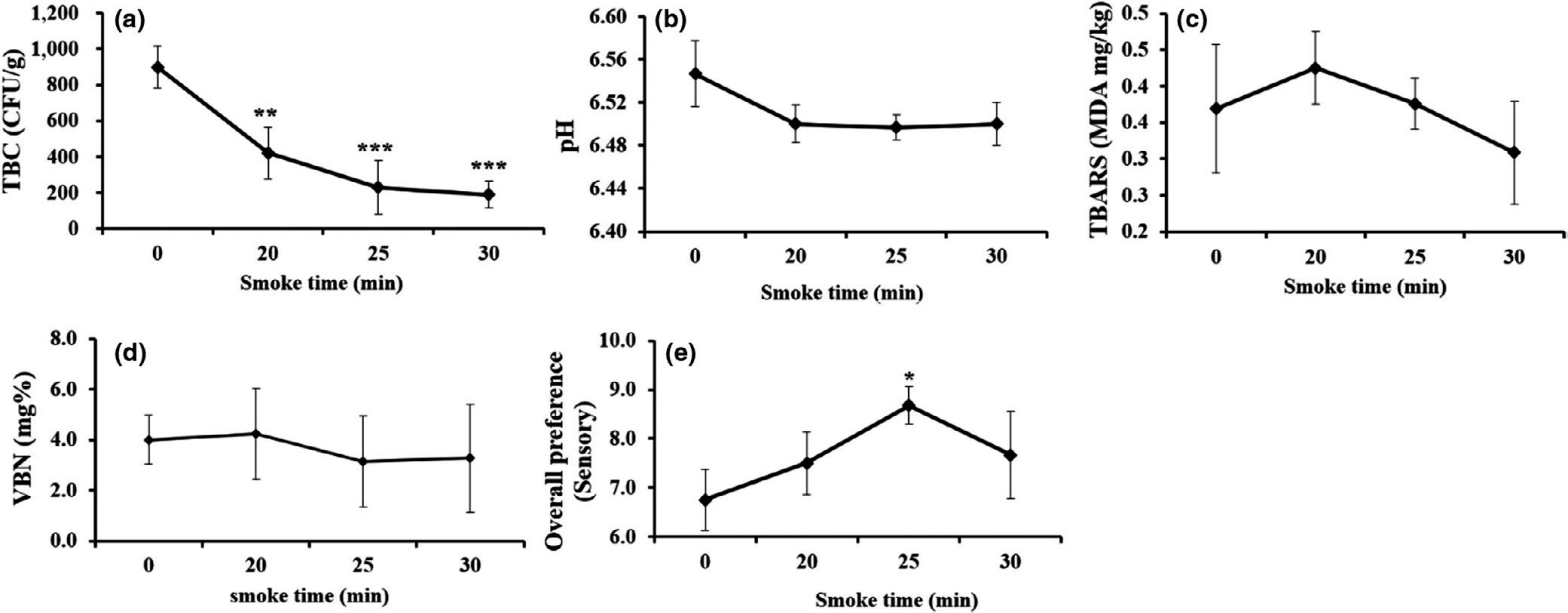
Effect of the best smoke time (0, 20, 25, and 30 min) on TBC (a), pH (b), TBARS (c), VBN (d), and overall preference (e) in hot‐smoked JSM. Data were expressed as mean ± std, and statistical significant level was expressed as *p < .05, **p < .01, and ***p < .001, compared with smoke time 0 (which is considered as raw JSM) (ANOVA)

The postharvest quality deterioration of fishery products is mainly because of the excessive level of bacterial growth to which, later on, the product becomes unsuitable and unsafe for consumption. In view of that, the total bacterial count (TBC) was taken consideration for quality assessment and, eventually, significantly (*p* < .01, .001) decreased owing to the increase of smoking time at 20, 25 and 30 min (Figure 4a).

#### 3.4.2 Effect of smoking time on pH

In the processing of fishery products, the pH is considered to be the one of the indicators for quality assessment due to its depletion effects during storage conditions. The smoking time whether has favorable influences on the pH quality, to prove that, the present study was found no initial pH changes at the smoking time of 20, 25, and 30 min (Figure 4b).

#### 3.4.3 Effect of smoking time on TBARS changes

The TBARS value is the result of degradation product of fats in a given food product, and it is expressed by measuring the malondialdehyde (MDA) content. As seen in the present study, a nonsignificant increase of MDA content at 20‐min smoking time, and then, a further nonsignificant decrease up to 30 min of smoking time in hot‐smoked JSM was observed (Figure 4c).

#### 3.4.4 Effect of smoking time on VBN changes

The volatile base nitrogen is the indicative component to measure the degree of spoilage of fish and fish products. The VBN level in hot smoked JSM was nonsignificantly decreased with increased smoking time; especially, it started from 25 to 30 min (Figure 4d). The range of VBN value in hot‐smoked JSM product was 4.00 ~ 3.28 (mg %).

#### 3.4.5 Effect of smoking time on overall preference (sensory)

Based on the trained panelists score, the overall preference was significantly (*p* < .05) increased at the smoking time of 25 min and then declined to its sensory quality at the smoking time of 30 min (Figure 4e).

### 3.5 Effect of hot smoking on storage condition of JSM in consecutive sampling days

#### 3.5.1 Effect of storage time on microbiological activity

The microbial activity in fishery products is greatly influenced by the temperature, especially while keeping at the storage condition. We maintained two different storage temperature at 10 and 15℃ of 25 min (optimized) smoked JSM, and thereafter, total bacterial count (TBC) and fecal coliform density of experimental JSM product were studied intermittently throughout the storage period from Day 0 to Day 42, as shown in Table 1. The results indicated that at 10℃ storage condition no TBC density was observed except at 40 days of sampling but at 15℃ storage condition TBC appeared as early at 25 days of storage period. No fecal coliform was observed with the days along with sampling at 10℃ until to 42 days of storage period but at 15℃ no coliform was visible with limited storage period to 32 days. Accumulated results suggested that the shelf life of smoked JSM could be extended at the storage condition of 10℃ as compared to 15℃.

#### 3.5.2 Effect of storage time on VBN changes

**FIGURE 5:**
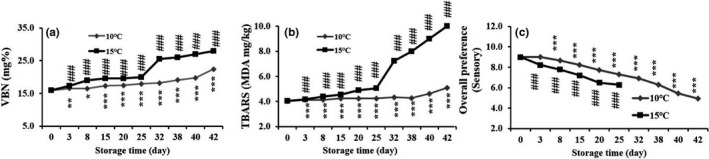
Effect of the storage time on VBN (a), acid value (b), and overall preference (sensory) (c). The quality features were compared at two different storage temperature (10 ℃ and 15 ℃) in the storage days of 0 to 42. Data were expressed as mean ± std, and statistical significant level was expressed as *p < .05, **p < .01, and ***p < .001, compared with the day 0 (ANOVA)

The smoking of JSM at 25 min (optimized) was kept in two different storage temperature such as 10℃ and 15℃ for 0 to 42 days. The VBN value was found to be significantly (*p* < .001) increased with the extension of the storage period (Figure 5a). From 0 to 42 storage days, all of the VBN values were in between the ranges of 16.0–24.4 (mg%) and 16.0–28.0 (mg%) at 10℃ and 15℃ temperature condition, respectively, indicating that at 10°C, it was suitable condition for storing the processed JSM.

#### 3.5.3 Effect of storage time on TBARS changes

With high peroxidizable lipid contents, fish and fishery products at different storage conditions are potentially vulnerable to quality loss. In two different storage temperature (10 and 15℃) for 0 to 42 days, the TBARS values were found to be significantly (*p* < .001) increased along with the times and remained in between the ranges of 4.0–5.1 mg/g and 4.0–10.0 mg/g, respectively. The results suggested that at 10℃ temperature storage condition for the processed at 25 min smoking JSM had shown to have more the acceptable limit of TBARS value and regarded as safe for consumers.

#### 3.5.4 Effect of storage time on overall preferences

In this study, sensory quality test has done within two‐storage condition explicitly at 10℃ and 15℃ temperature. The sensory score of hot‐smoked JSM with an optimum smoking time of 25 min was decreased with the increase in storage time. Based on panelist score, at 10℃ storage condition of smoked JSM remained safe and consumable even up to 42 days. On the other hand, 15℃ of storage condition was found to be acceptable and remained safe and eatable up to 25 days, and over this period, it declined its sensory quality (Figure 5c).

### 3.6 Effect of hot smoking of JSM on TMAO changes

**FIGURE 6:**
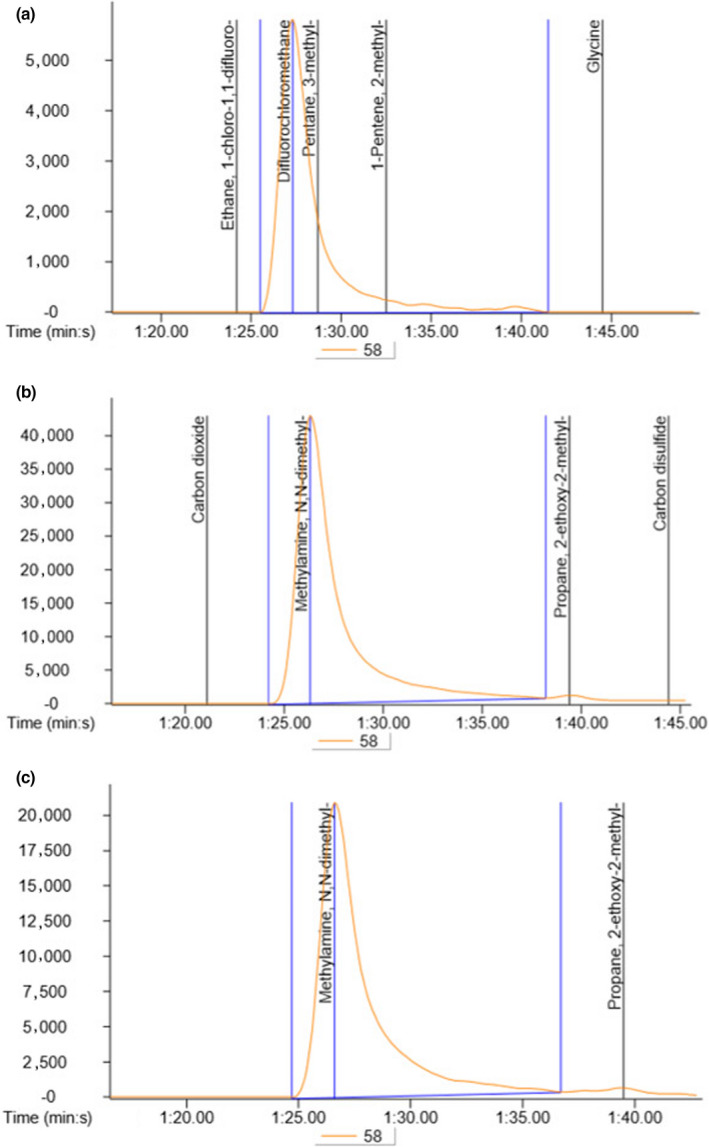
The GC/MS profiles for identification and quantification of trimethylamine N‐oxide (TMAO) level in comparing with the standard TMAO (a), raw JSM (b), and hot‐smoked JSM

Many of the spoilage bacteria inhabited on fish can effectively utilize TMAO as an electron acceptor in anaerobic respiration, as a by‐product, TMA is produced, and it is an important indicator of fish spoilage. As results obtained, TMAO content in both raw and processed JSM was shown to have 5.89 ± 1.19 μg and 1.63 ± 0.26 per 100 g of sample, respectively, as confirmed by GC/MS analysis, indicating that smoked JSM had suppressive effects on TMAO changes (Figure 6).

### 3.7 Effect of hot smoking of JSM on nutritional quality changes

The nutritional quality of processed fish or fishery product may differ during different processing treatments. The calories value was estimated at 141.625 kcal/100 g in hot‐smoked JSM. The proximate composition such as carbohydrate, lipid, and protein content in processed JSM was 0.512, 2.353, and 29.600 g/100 g, respectively (Table 2). The amount of mineral content such as sodium, potassium, calcium, and iron in hot‐smoked JSM was 211.792, 531.561, 43.224, and 0.407 mg/100 g, respectively. The present study revealed that the hot‐smoked JSM exerted higher nutritional value along with improved physicochemical properties and antimicrobial activities, which together offer to be the premium quality of processed food products for consumers.

### 3.8 Effect of hot smoking of JSM on fatty acid changes

The fatty acid composition was analyzed in hot‐smoked JSM fish products. The overall results of the fatty acid content indicated the higher level of monounsaturated fatty acids (MUFAs; 7.78 g/100 g) as followed by polyunsaturated fatty acids (PUFAs; 7.03 g/100 g) and saturated fatty acids (SFA; 6.30 g/100 g) in processed JSM. Moreover, the predominant fatty acid in JSM was reported to be oleic acid (OA; 5.79 g/100 g) as followed by docosahexaenoic acid (DHA; 4.19 g/100 g), eicosapentaenoic acid (EPA; 1.82 g/100 g), and palmitoleic acid (PA; 1.46 g/100 g). The results revealed that hot‐smoked treatment of JSM had increasing effects on the fatty acid content, most particularly, DPA and EPA (omega‐3). Therefore, the hot smoking of JSM product caused high fatty acid contents may be explained by the fact that most of the beneficial fatty acids remained stable or unchangeable during processing, as seen in the Table 3.

## 4 DISCUSSION

Fish and other aquatic animals are not only being used as tasty substances of daily diet either in raw or fishery products but also they are nutritionally and functionally enriched, of which, the plethora of protein, lipids, useful secondary metabolites, and many other essential vitamins and minerals are becoming a subject of consumer attention (Sampels, 2015). Fish is a difficult culinary object, because it is easily oxidized, developed off‐flavor, and subsequently spoiled when marketed. Japanese Spanish Mackerel, *Scomberomorus niphonius*, is considered as one of the important commercial fish species in South Korea and being marketed as a raw or frozen state. Currently, the appropriate handling and preservation technique of JSM during the postharvest processing to the consumer plate are not clearly understood. The main objective of the present study is to develop an effective processing and preservation technique and, subsequently, determine the sensorial, physicochemical, and microbial changes at different storage conditions, of which a premium quality of hot‐smoked JSM will be ensured.

Among different preservation techniques, hot smoking has an especial appeal to the consumer due to the fact of its uniqueness of sensorial properties. It is evident that the sensory quality of the fish product is significantly enhanced by the use of different sawdust materials of plant origin while smoking (Küçükgülmez, Eslem Kadak, & Celik, 2010), because a rich source of volatile compounds preserved, among which phenols are predominantly responsible compound to develop color and aroma to the food materials (Dillon, Patel, & Martin, 1994). When the hot smoke (70℃) with different sawdust materials was employed with maintaining the different smoke time (0, 20, 25, and 30 min) to process JSM fillet, the higher sensory attributes such as appearance, odor, taste, color, texture, and overall preferences were achieved with an optimized smoking time of 25 min. Küçükgülmez et al. (2010) found his study that significant sensorial impacts had been observed when working on hot‐smoked Wels catfish. A similar study was performed by our previous study that oak sawdust was considered as one of the most preferred smoking materials when smoked on the adductor muscle of pen shell *Atrina pectinate *(Mohibbullah, Won, et al., 2018). In the case of developing consumer products, the 9‐point hedonic scale method has proven itself as a reliable, effective, and simple measuring tool (Lim, 2011). Moreover, sensory evaluation is a rational nature of judgments by panelists, in where the best categories of sensory attributes can be achieved.

The food texture is generally correlated with the oral sensory perception. The texture quality and weight loss of hot‐smoked JSM were remained unchanged until 20 min of smoking time but gradually increased in the next 25 and 30 min; however, in the case of hardness, it did not increase significantly. In compared with the results of weight loss, it might be the cause of water loss from JSM and the reason the firmness of texture was increased to have good sensorial impacts on consumer (Oz, Ulukanli, Bozok, & Baktemur, 2015). The color and odor interact with each other while taking the decision, of important attributes to the consumers, whether accepted or rejected it when coming closer to the foods (Nollet & Toldrá, 2009). The smoking time and temperature did not influence the color of JSM, which has been in accord with the previous study by Zzaman, Bhat, Yang, and Easa (2017).

The smoke of sawdust acted as an antibacterial agent, which has been well known to prevent bacterial growth (Bashir et al., 2017; Goulas & Kontominas, 2005; Guillén & Errecalde, 2002). Chen et al. (2015) have found similar results as bacterial contamination can be prevented in the frozen storage condition. With the optimum time for smoking at 25 min, JSM showed relatively the best suppressing effect on TBC. Moreover, similar positive consequences were observed as seen in smoked JSM maintained desirable pH, lowered MDA content, and reduced level of VBN. The VBN value remains acceptable range in hot‐smoked JSM in comparison with the reference VBN value of fresh fish 5–10 mgN/100 g muscle (Huss, 1988). The previous study supported the present study as smoking significantly decreased the VBN level of processed squid (Sutikno et al., 2019). The acceptable range of TBARS is between 7 and 8 MDA mg/kg, whereas some others suggested being not more than 5 MDA mg/kg (Oğuzhan Yildiz, 2015), and therefore, those parameters were far below level in the smoked JSM, respectively, as compared to the reference value. Moreover, the smoked JSM product also extended shelf life up to 42 days at 10 ℃ of storage temperature, as confirmed by VBN value, MDA content, sensorial preference, TBC, and coliform density. While other studies reported that the sensory scores of smoked catfish and mackerel decreased with the increase of storage time, and the product remained safe up to 16 days and became unfit for human consumption on day 24 (Kolodziejska, Niecikowska, Januszewska, & Sikorski, 2002; Yanar, 2007).

In death fish, bacterial action takes place that is converted TMAO to TMA (trimethylamine), which produces off‐odor and does not taste sweet in fish (Venugopal, 2005; Nollet & Toldrá, 2009). Thus, the determination of TMAO content in fishery products is one of the approaches to evaluate the degree of fish spoilage. In our study, the smoked JSM had promising effects in reducing the content of TMAO significantly, as compared to raw fish. This level of TMAO was considered to be safe for human consumption (Mohibbullah, Won, et al., 2018). In the nutritional aspects, the chemical composition of fishery products varies greatly in relation to age, sex, environment, season, and even among the same species. Since, hot smoking is associated with drying, cooking, and deposition of chemicals of wood smoke, in which the proportions of overall nutrients are increased due to the fact of decreasing moisture content from the smoked product. The proximate composition of protein, lipid, minerals, and vitamins was found to be comparable with those of the previous study, in order to meet the food regulations (Aremu, Namo, Salau, Agbo, & Ibrahim, 2013). In fatty acid facts of smoked JSM, EPA (20:5 n‐3) and DHA (22:6n‐3) were present highest level in PUFAs, of which, however, MUFAs exceptionally were higher than that of PUFA in our study. The results were similar to that of the previous study (Alasalvar, Miyashita, Shahidi, Wanasundara, and (Eds.)., 2011). The PUFAs including omega‐3 fatty acids such as EPA and DHA have been reported to be effective in neurodegenerative complications, cardiovascular diseases, arthritis, and mood stabilizers for bipolar disorder (Breslow, 2006; Mohibbullah, Choi, et al., 2018; Mohibbullah et al., 2015).

## 5 CONCLUSION

In conclusion, the hot‐smoked (70℃) processing technique with oak sawdust improved the physicochemical, microbiological, and sensorial attributes of JSM. The trimethylamine N‐oxide (TMAO) was found to be suppressed by the processed JSM. Contemporarily, it contained higher nutritional value with a higher percentage of PUFAs including EPA and DHA followed by MUFAs. Therefore, due to the improved sensorial, physicochemical, and microbial attributes, the hot‐smoked JSM extended shelf life up to 42 days at 10℃ of storage temperature condition. This technique can be useful for the processing and preservation of Japanese Spanish mackerel to meet the food demand, specification, and regulations in the emerging fish market.

## CONFLICT OF INTEREST

The authors declare no conflict of interest.

## ETHICAL STATEMENT

Not applicable.

6

**TABLE 1:** Microbial quality of the hot‐smoked JSM in storage condition

Storage time (Days)	TBC (CFU/g)		Coliform	
	10℃	15℃	10℃	15℃
0	0	0	0	0
3	0	0	0	0
8	0	0	0	0
15	0	0	0	0
20	0	0	0	0
25	0	30	0	0
32	0	50	0	0
38	0	–	0	–
40	0	–	0	–
42	10	–	0	–

**TABLE 2:** Nutritional value of the hot‐smoked JSM

Test items	Unit	Test results
Calories	Kcal/100 g	141.625
Sodium	mg/100 g	211.792
Carbohydrate	g/100 g	0.512
Sugars	g/100 g	ND
Crude fat	g/100 g	2.353
Trans fat	g/100 g	0.002
Saturated fat	g/100 g	0.635
Cholesterol	mg/100 g	80.249
Crude protein	g/100 g	29.600
Potassium	mg/100 g	531.561
Calcium	mg/100 g	43.224
Iron	mg/100 g	0.407
Vitamin D	mg/100 g	ND

**TABLE 3:** Fatty acid composition of the hot‐smoked JSM.

Fatty acid	Mackerel (g/100 g)
Butyric acid	0.00
Caproic acid	0.00
Caprylic acid	0.00
Capric acid	0.00
Lauric acid	0.00
Tridecanoic acid	0.00
Myristic acid	0.88
Pentadecanoic acid	0.10
Palmitic acid	4.03
Stearic acid	0.95
Elaidic acid	0.00
Margaric acid	0.21
Heneicosanoic acid	0.01
Behenic acid	0.03
Arachidic acid	0.06
Tricosanoic acid	0.00
Lignoceric acid	0.03
∑ SFA	6.30
Myristoleic acid	0.01
Pentadecenoic acid	0.00
Palmitoleic acid	1.46
Oleic acid	5.79
Cis−11‐Eicosenoic acid	0.35
Erucic acid	0.06
Nervonic acid	0.11
∑ MUFA	7.78
Linolelaidic acid	0.00
Linoleic acid	0.27
Margaroleic acid	0.00
ϒ‐Linlenic acid	0.02
Cis−11, 14‐Eicosenoic acid	0.23
Cis−11, 14, 17‐Eicosenoic acid	0.02
α‐ Linlenic acid	0.15
Dihomo ϒ‐Linolenic acid	0.04
Arachidonic acid	0.29
Docosadienoic acid	0.00
Eicosapentaenoic acid	1.82
Docosahexaenoic acid	4.19
∑ PUFA	7.03

MUFA, Monounsaturated fatty acid; SFA, Saturated fatty acid; PUFA, Polyunsaturated fatty acid.
